# Intra-Articular Umbilical Cord Derived Mesenchymal Stem Cell Therapy for Chronic Elbow Osteoarthritis in Dogs: A Double-Blinded, Placebo-Controlled Clinical Trial

**DOI:** 10.3389/fvets.2019.00474

**Published:** 2019-12-20

**Authors:** Stanley E. Kim, Antonio Pozzi, Jiunn-chern Yeh, Mariana Lopez-Velazquez, Jo Anne Au Yong, Sarah Townsend, Anna E. Dunlap, Scott A. Christopher, Daniel D. Lewis, Matthew D. Johnson, Kathryn Petrucci

**Affiliations:** ^1^Department of Small Animal Clinical Sciences, College of Veterinary Medicine, University of Florida, Gainesville, FL, United States; ^2^Animal Cell Therapies, San Diego, CA, United States

**Keywords:** degenerative joint disease, regenerative therapy, canine, lameness, force-plate

## Abstract

**Background:** Intra-articular stem cell therapy may help alleviate lameness caused by osteoarthritis in dogs. Umbilical cord-derived stem cell (UMSC) therapy has not yet been investigated in a veterinary clinical study. We hypothesized that dogs treated with intra-articular UMSC will have improved limb function and quality of life when compared to dogs treated with a saline placebo injection.

**Methods:** This was a prospective, double-blinded, placebo-controlled clinical trial in client-owned dogs with chronic elbow osteoarthritis with a follow-up time of 6 months. Dogs were assigned to receive intra-articular UMSC (*n* = 38) or a saline placebo intra-articular injection (*n* = 30). Outcome measures included the Canine Brief Pain Inventory score (CBPI) and peak vertical force (PVF) from force-platform gait analysis. Treatment was considered successful when there was a decrease in the Pain Severity Score of at least one and a decrease in the Pain Interference Score of at least one from baseline. Success rates and PVF were compared between groups.

**Results:** No adverse effects associated with UMSC were noted. Of the dogs completing the study, treatment success in the UMSC (*n* = 28) vs. placebo groups (*n* = 23) was observed in 54 vs. 28% of dogs at 1 month, 50 vs. 27% at 3 months, and 46 vs. 14% at 6 months, respectively. Success rate in the UMSC group was significantly higher than the placebo group at 1 and 6 months after treatment. However, no differences in PVF of the affected limb over time was observed in either group.

**Conclusions:** Intra-articular UMSC for osteoarthritis may improve clinical signs based on owner observations.

## Introduction

Osteoarthritis (OA), or degenerative joint disease, is the most common cause of chronic pain affecting dogs in the United States (1). Adult articular cartilage has limited regenerative capability, which makes OA a progressive disease and challenging to treat. The goal of OA treatment is to reduce pain and increase limb function while improving quality of life. Common conventional non-surgical treatment options include the administration of non-steroidal anti-inflammatory drugs and poly-sulfated glycosaminoglycans, as well as nutritional and behavior modifications. Many of these therapeutics are thought to alleviate arthralgia by mitigating inflammation. These conventional treatment modalities have limited efficacy and duration of action, are not curative, and can be associated with adverse effects (2).

Mesenchymal stem cells (MSC) have been investigated for the treatment of OA in dogs (3–13). While the precise mechanism of action is unknown, the therapeutic effect of MSC for OA is predominately attributed to their anti-inflammatory properties (14). Studies evaluating the efficacy of adipose derived and bone marrow derived MSC delivered intra-articularly in animal models of induced and spontaneous OA have shown improvements through various outcome measures (6–11), with encouraging results for the application of MSC for the treatment of OA in a clinical setting (3–5, 7–10). Black et al. found subjective improvements in lameness, pain, and range of motion in dogs with elbow and hip OA treated with intra-articular autologous stromal vascular fraction containing adipose derived MSC (3, 4). More recently, Vilar et al. reported a significant increase in peak vertical force (PVF) and vertical impulse (VI) on force plate analysis in dogs with hip OA 6 months following treatment with intra-articular autologous adipose-derived MSC (9, 10). However, most of the studies investigating MSC have several limitations including lack of randomization or control group, small sample size and/or only subjective outcome measures (3–5, 9, 10).

Most of the commercially available stem cell therapies in dogs are autologous, and therefore require retrieval of tissue or fluids from the subject undergoing treatment. This multi-step process creates a time lag between the decision to treat and delivery of stem cells. More importantly, harvest of stem cells typically requires surgery and is therefore associated with additional morbidity and cost. These limitations can be overcome by using allogenic MSC, which could be considered as being available “off-the-shelf.” In a recent study, intra-articular administration of allogenic adipose derived MSC was considered efficacious when compared to a saline placebo in dogs with OA (7). An alternate source of MSC is the umbilical cord. Umbilical cord derived MSC (UMSC) have the advantage of utilizing “waste” tissues that are collected during a cesarean section planned for reasons unrelated to UMSC harvest (12, 15). Additional advantages of UMSC over MSC sourced from other sites include acquisition of younger cell types that have a higher capacity to proliferate with wide differentiation potential (15). While UMSC have several promising theoretical advantages, the risks and overall therapeutic response when used for treating OA in dogs are unknown.

The aims of this study were to evaluate the efficacy and safety of intra-articular allogeneic UMSC for the treatment of chronic elbow OA in dogs. We hypothesized that dogs treated with intra-articular UMSC would have improved limb function and quality of life when compared to dogs treated with a saline placebo injection.

## Materials and Methods

This study was a prospective, double-blinded, placebo-controlled clinical trial at the University of Florida Small Animal Hospital. All procedures were approved by the University of Florida Institutional Animal Care and Use Committee, as well as the Veterinary Hospital Research Review Committee.

### Selection Criteria

Client-owned dogs with clinical signs of elbow OA were considered for enrollment. Owner consent was obtained prior to screening; the consent form detailed the study protocol including the screening procedures. To be eligible for inclusion, dogs had to be >12 months and <11 years of age, weigh between 13 and 60 kg, have a visibly apparent unilateral forelimb lameness upon examination by a board-certified veterinary surgeon, and have a chronic (≥6 months) history of forelimb lameness. Lameness must have been attributable to elbow OA based on orthopedic examination by a board-certified veterinary surgeon, including pain or resistance on elbow manipulation, as well as evidence of OA on diagnostic imaging. Owners completed the Canine Brief Pain Inventory (CBPI) survey [(15), https://www.vet.upenn.edu/research/clinical-trials-vcic/our-services/pennchart/cbpi-tool/cbpi-tool-form] and they must have reported both the baseline pain severity (PSS) and pain interference (PIS) scores of ≥2 for their dog.

The presence of osteoarthritic changes in the elbow(s) was confirmed by CT imaging of the forelimbs from mid-humerus to mid-radius and ulna. For CT imaging, dogs were sedated with dexmedetomidine at 10–15 μg/kg intravenously; during image acquisition, dogs were positioned in dorsal recumbency with the forelimbs extended cranially, and both elbows were scanned at the same time. Each case was assessed for the presence of unilateral or bilateral elbow OA on CT images; the degree of OA was not graded, but only assessed as being present or absent. Underlying pathology such as medial coronoid disease, radio-ulnar incongruency, osteochondritis was identified and recorded. Confirmation of general good health was achieved with a complete physical examination by a veterinarian, review of medical history, and clinical pathology evaluations (CBC, serum biochemistry profile, and urinalysis). Dogs on neutraceuticals and/or medications such as non-steroidal analgesic drugs (NSAID) must have been on a stable dose for at least 4 weeks prior to the start of the study, and throughout the study duration. Dogs were excluded if they had forelimb lameness attributable to disease(s) other than elbow OA, hindlimb lameness of any cause, cranial cruciate ligament insufficiency, neurologic disease, malignancy, septic arthritis, current administration of tetracycline antibiotics (due to possible immunomodulatory effects), any febrile disease, immune mediated disease, or other concurrent systemic disease that might limit the dog's lifespan. Dogs with bilateral elbow OA were considered candidates for the study if lameness was unilateral or if there was an asymmetric bilateral forelimb lameness.

At the initial evaluation and all rechecks, force plate evaluation was performed at a trot (16). Gait analysis was performed at trot because lameness may be more apparent at the trot rather than walking. Approximately 15–20 passes of combined left and right forelimbs were collected, and the most representative 5 passes per limb were analyzed. Asymmetry in PVF of ≥4 N/kg between forelimbs on force-plate analysis must have been evident the time of screening for inclusion into the study; the side of lameness on force plate analysis must also have corresponded to the owner's observation of the most affected side.

### Treatment Allocation

Dogs were allocated into either UMSC or placebo groups according to the method of minimization. Because true randomization requires very large numbers, the method of minimization is often used to stratify groups in clinical trials (17). The method of minimization requires identifying several factors that are most likely to affect the outcomes of the study and attempts to balance these factors equally across the two groups. In this trial, the factors considered were the degree of lameness based on force-plate evaluation, the use of non-steroidal anti inflammatories, body condition score, and age. The study coordinator, who was a veterinarian, determined the allocation of dogs based on these factors in the order listed above. In dogs with bilateral lameness, only the clinically worse elbow was injected.

### Blinding

The treatment allocation was known only to the study coordinator. The intra-articular treatment was performed by the study coordinator in the absence of any individual that was involved in case evaluations. The owners, all other veterinarians, and all clinic personnel who were involved in the study were blinded to treatment group.

### Stem Cell Collection and Preparation

Canine umbilical cords were collected at veterinary hospitals from healthy donor puppies who met Animal Cell Therapies' donor eligibility criteria at the time of cesarean section in an aseptic manner. Dams must have been considered systemically healthy upon physical examination, current on vaccinations (Rabies and Distemper), negative and on preventative for heartworm disease, negative for occult infectious diseases including *Anaplasma* spp., *Babesia* spp., *Bartonella* spp., *Brucella canis*, Canine Hemotropic Mycoplasma, *Ehrlichia* spp., and *Leishmania* spp. based on PCR on serum. The placenta and umbilical cords were placed in cold sterile transport medium and sent to the processing facility. Umbilical cords were processed according to the U.S. Food and Drug Administration (FDA) Good Tissue Practices, under sterile conditions. The cells were mainly derived from Warton's jelly. Briefly, using sterile instruments and technique, the cord tissues were washed until mostly blood-free. After removing the remaining placentas and major blood vessels, the cord tissues were digested with an enzyme solution. After enzymatic digestion, the cells released from the tissues were pelleted by centrifugation and seeded on cell culture vessels. Cells were cultured with proprietary culture medium and maintained using standard cell culture techniques. Each 0.5 ml dose contained approximately 7 million cells.

Master and working cell banks were generated according to Animal Cell Therapies SOPs under the FDA Current Good Manufacturing Practices. The UMSC were tested for mycoplasma, endotoxin, fungal, aerobic and anaerobic bacteria, and cell viability was quantified prior to lot release. Additionally, after processing, the passage one cells were further tested for canine blood donor pathogens using PCR. The cells were characterized to conform to Animal Cell Therapies' standard growth curves, tri-lineage (adipocyte, osteocyte, and chondrocyte differentiation) potency assays and cell surface marker expression. Positive markers for canine umbilical cord tissue derived MSC analyzed by flow cytometry included CD44, CD90, CD105, and negative markers included CD45, CD34, CD14, CD19, and MHC-II. Prior pilot investigations (unpublished data) found cell viability before shipping of 98%, and average cell viability of 92% (ranged from 88 to 95%) at the destination (cell viability was determined roughly 24 h after packing).

### Treatment

Dogs underwent treatment with either intra-articular UMSC therapy (UMSC group) or placebo injection using 0.9% NaCl (placebo group) within 2 weeks of screening and recruitment. For all dogs, sedation with dexmedetomidine at 10–15 μg/kg intravenously was performed for the purposes of arthrocentesis and joint injection. After aseptic preparation, arthrocentesis was performed in a sterile manner at the mid-medial aspect of the elbow joint; <0.5 ml of the joint fluid was removed and then evaluated cytologically to ensure it was consistent with OA.

All treatments (placebo and UMSC) were kept on ice until 5 min prior to injection, then thawed at room temperature. The UMSC suspension was gently agitated before being drawn into a sterile syringe. The preplaced 22 g hypodermic needle used for arthrocentesis was used to administer 0.5 ml of UMSC suspension or 0.5 ml of sterile saline. Sedation was reversed with atipamezole at 100–150 μg/kg administered intramuscularly.

Owners were advised to monitor the treated joint for signs of infection or inflammation (redness, swelling, discharge, acutely worsening lameness, lethargy). Owners were also advised to restrict their dog's activity for 2–3 days following joint injection. If no signs of discomfort were noted, dogs were permitted normal activity. Dogs were withdrawn from the study at any time due to unacceptable outcomes such as deterioration of limb use and/or the development of a serious adverse event. Dogs in the placebo group were eligible for an intra-articular dose of UMSC at their 6 months evaluation or at the time of withdrawal.

### Outcome Measures

Data was scheduled for collection immediately prior to treatment (day 0), then at day 30, 90, and 180 following treatment. At each time point, owners scored the level of pain with the CBPI (18), and degree of lameness with the Hudson Visual Analog Scale (HVAS) (19). Owners and evaluators did not see their prior scores when completing the new scoring forms. Dogs also underwent force-platform gait analysis as described above, and veterinary orthopedic examination. For force-platform gait analysis, the symmetry index was calculated according to the formula (20):

$$\begin{array}{l} \text{Symmetry Index}=\left(0.5\times \left[{\text{PVF}}_{\text{a}}-{\text{PVF}}_{\text{c}}\right]\right)/\left({\text{PVF}}_{\text{a}}-{\text{PVF}}_{\text{c}}\right)\times 100\%, \end{array}$$

where PVF_a_ = mean peak vertical force of the affected fore limb, and PVF_c_ = mean peak vertical force of the contralateral fore limb. The CBPI questionnaire is a validated two-part instrument: the PSS is the mean of four items scored on an 11-point (0–10) numerical scale, and the PIS is the mean of 6 items scored similarly (18). In addition, the CBPI includes a single question for the owner to rate his or her overall impression of the dog's quality of life over the last 7 days using the following terms (owner chose one): poor, fair, good, very good, or excellent. The HVAS questionnaire, another validated clinical outcomes tool, required owners to mark the degree of pain and lameness on a 10 cm horizontal line with positive and negative descriptors of lameness provided at opposing ends of the scale (19). Orthopedic assessment was performed by a board certified veterinary surgeon. Lameness was evaluated at a walk, trot, and standing. The surgeon evaluated joint effusion, muscle mass, range of motion, crepitus, and remodeling (joint thickening) by direct visualization and palpation on a scale of 0–5 (0 = normal, 1 = mild, 2 = mild to moderate, 3 = moderate, 4 = moderate to severe, and 5 = severe).

### Adverse Events

Safety was monitored through the development of adverse events throughout the study period. Any adverse event following treatment was recorded, regardless of its suspected relationship to the treatment. Serious and non-serious adverse events, defined according to FDA guidelines, were included (21). Non-serious events were further qualified as mild (not interfering with the dog's usual function), moderate (interfering somewhat with the dog's usual function), and severe (interfering significantly with the dog's usual function). Adverse events were assessed in regards to their suspected relationship to the treatment, and were classified as definite (distinct temporal relationship between the treatment and the known reaction), probable (reasonable temporal relationship between the treatment and the reaction), possible (reasonable temporal relationship between the treatment and the reaction, but the reaction could also be explained by the patient's clinical state or other factors), unlikely (poor temporal relationship between the treatment and the reaction, and the reaction is easily explained by other factors), or unrelated (the event occurred prior to the treatment, or the reaction can be completely explained by other factors).

### Statistical Analysis

Based on a pre-study power analysis using results from a pilot field study at our institution, 25 dogs per treatment group was found to provide >90% power with α = 0.05 in detecting a difference in treatment success rate of 20% based on CBPI (as defined below). In order to account for attrition during the study, we aimed to recruit 30 dogs per group.

Safety was assessed in the intent-to-treat (ITT) population, which included all dogs that received treatment (UMSC and placebo). The rate and type of adverse events were noted and descriptively compared for each treatment group.

The UMSC and placebo group populations were assessed for differences in pre-treatment variables with an unpaired *t*-test for age, orthopedic score, CBPI, and HVAS (all normally distributed according to Shapiro-Wilks test), and with a Chi-Squared test for NSAID use.

Efficacy was determined from the Per Protocol Population (PPP), which included dogs without major protocol violations. The primary variable used to assess efficacy was based on the CBPI. For each dog, treatment success was defined for post-treatment time points as: (1) a decrease in PSS of at least one from baseline, and (2) a decrease in PIS of at least 1 from baseline (18). Any dog that did not meet this criterion for success was considered a failure. Failure was carried forward, where each dog assessed as a treatment failure at a post-treatment time point was considered as a treatment failure at subsequent time points. Dogs that were withdrawn from the study as the result of perceived lack of efficacy by owner or veterinarian were also considered as treatment failure. Success rates were compared between groups at each time point with a Chi-squared test.

Secondary outcome variables compared between groups in the PPP at each time point were: sum indexed orthopedic examination scores, sum indexed HVAS scores, and PVF of the affected limb. Comparisons were performed with a repeated measures analysis of covariance, with baseline values as a covariate. For all statistical analyses, *P* < 0.05 was considered significant.

## Results

### Animals

**Figure 1:**
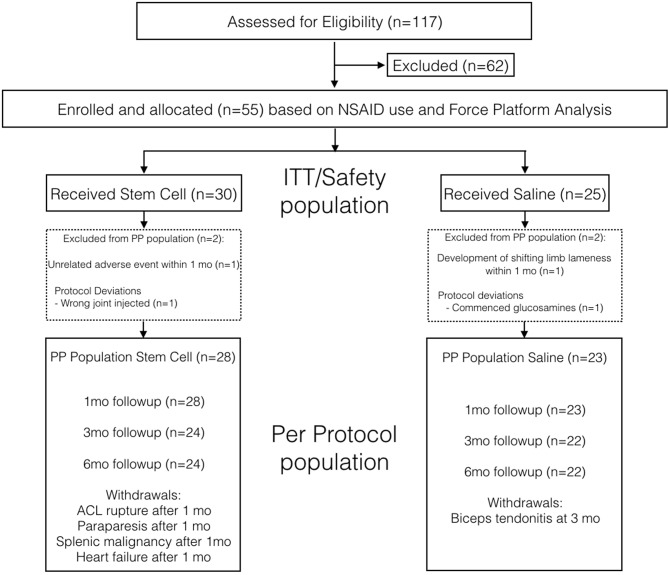
Case flow indicating how the safety (intent to treat) and per protocol populations were identified for the UMSC and placebo treatment groups.

One-hundred and seventeen dogs were assessed for eligibility, of which 55 dogs met the inclusion criteria and were enrolled into the study (Figure 1). The main reasons for initial exclusion were lack of lameness (*n* = 15), pelvic limb lameness (*n* = 12), and other cause of forelimb lameness (*n* = 8). In the ITT population, 30 dogs were allocated to the UMSC group, and 25 dogs were allocated to the placebo group. There were two dogs in the UMSC group that did not qualify for the PPP: one dog developed gastrointestinal perforation due to multiple concomitant NSAID administration that was not prescribed by a veterinarian at 30 days from treatment, and the wrong joint was injected in one dog. There were also two dogs in the placebo group that did not qualify for the PPP: one dog had unexplained and frequent shifting forelimb lameness noted in the first month after treatment, and one dog was administered Adequan in the first month after treatment. Therefore, a total of 51 dogs were included in the PPP, with 28 dogs in the UMSC group and 23 dogs in the placebo group. There was no significant difference in age, body weight, proportion of dogs with NSAID use, and symmetry index between groups in the PPP (Table 1).

**Table 1:** Pre-treatment variables of the per protocol population.

	**Treatment arm**			
**Parameter**	**Placebo (*N* = 23)**	**UMSC (N = 28)**	**All subjects (*N* = 51)**	***P-*value**
**Gender—*****n*** **(%)**				
Male	14 (60.9%)	14 (50.0%)	28 (54.9%)	0.44
Female	9 (39.1%)	14 (50.0%)	23 (45.1%)	
**Age (y)**				
Mean (SD)	6.2 (3.3)	7.2 (2.6)	6.7 (3.0)	0.26
Median	7.0	8.0	7.0	
Min, Max	1.0, 11.0	2.0, 11.0	1.0, 11.0	
**Weight (kg)**				
Mean (SD)	29.7 (9.0)	32.6 (8.3)	31.3 (8.7)	0.23
Median	30.7	31.9	31.8	
Min, Max	11.5, 49.5	19.2, 59.1	11.5, 59.1	
**Body condition score**				
5	9 (39.1%)	12 (42.9%)	21 (41.2%)	0.16
6	7 (30.4%)	9 (32.1%)	16 (31.4%)	
7	4 (17.4%)	7 (25.0%)	11 (21.6%)	
8	3 (13.0%)	0 (0.0%)	3 (5.9%)	
**Concurrent Medications**				
Yes	15 (65.2%)	16 (57.1%)	31 (60.8%)	0.56
No	8 (34.8%)	12 (42.9%)	20 (39.2%)	
**Symmetry index**				
Mean (SD)	−5.8 (3.5)	−6.5 (4.6)	−6.2 (4.2)	0.57
Median	−5.7	−5.5	−5.6	
Min, Max	−12.9, −0.2	−20.3, −0.3	−20.3, −0.2	

Within the PPP, bilateral elbow OA was evident on CT in 26/28 dogs (93%) and 17/23 dogs (74%) in the UMSC and placebo groups, respectively; there was no significant difference in the frequency of bilateral elbow OA between groups (*P* = 0.642). Based on CT findings, medial coronoid disease was suspected as the underlying disease process in all elbows with OA.

### Safety

No dogs in either group developed worsening of lameness following treatment that warranted removal from the study. Acute worsening of lameness was seen in one dog in the placebo group at 11 days following saline injection; however, the degree of lameness was not considered severe enough for either the owner or veterinarian to withdraw the dog from the study. Joint fluid cytology in that dog was consistent with OA, and the dog's exacerbation of lameness resolved after a 1 week period of rest. No other local adverse effects were noted in any dog, including any worsening of pain and swelling, or discharge from the treated elbow.

Serious adverse events occurred in five dogs in the UMSC group, and two dogs in the placebo group (Table 2). All of the serious adverse events were classified as unlikely to be related to treatment. One of the dogs in the UMSC group was excluded from the PPP because the serious adverse event occurred prior to the day 30 evaluation. The serious adverse events in the other four dogs of the UMSC group necessitated withdrawal of the dogs from the study between the day 30 and day 90 evaluation. A single dog in the placebo group was withdrawn from the study at the day 90 evaluation due to the development of clinically significant bicipital tenosynovitis.

**Table 2:** Adverse events.

**Treatment group**	**Time since treatment (days)**	**Type of event**	**Concomitant medications**	**Differential diagnosis**	**Estimated relationship to treatment by Veterinarian**	**Outcome**
UMSC	110	Collapse, ascites	Rimadyl 75 mg BID	Right sided congestive heart failure, portal hypertension, thromboembolic event.	Unlikely related to treatment	Humane euthanasia
UMSC	32	Acute abdomen	Previcox 227 mg SID Metacam 7.5 mg BID	Septic peritonitis (confirmed)	Unlikely related to treatment	Humane Euthanasia
UMSC	75	Hemoabdomen secondary to splenic mass	None	Haematoma, hemangioma, hemangiosarcoma	Unlikely related to treatment	Humane Euthanasia
UMSC	310	Peripheral lymphadenomegaly	None	Lymphoma (confirmed)	Unlikely related to treatment	Treatment with prednisone
UMSC	45	Cranial cruciate ligament rupture	None	Cranial cruciate Ligament Rupture (confirmed)	Unlikely related to treatment	Treatment with NSAIDs (surgery declined)
Placebo	91	Contralateral forelimb lameness	None	Bicipital tenosynovitis (confirmed)	Unlikely related to treatment	Treatment with rest, NSAIDs
Placebo	153	Nasal planum mass	Gabapentin, previcox, tramadol	Squamous cell carcinoma (confirmed)	Unlikely related to treatment	Nasal planum resection

### Owner-Assessed Outcomes

Treatment success based on CBPI at 1 month was observed in 15/28 UMSC dogs (54%) and 6/23 placebo dogs (28%); Treatment success based on CBPI at 3 months was observed in 12/24 UMSC dogs (50%) and 5/23 placebo dogs (27%) (Table 3). Treatment success based on CBPI at 6 months was observed in 11/24 UMSC dogs (46%) and 3/23 placebo dogs (14%). The treatment success rate in the UMSC group was significantly higher than the placebo group at 1 and 6 months after treatment, but no difference (*P* = 0.056) was observed at 3 months.

**Table 3:** Success rates based on CBPI results.

	**1 month**	**3 months**	**6 months**
Sample sizes (stem cell group, placebo group)	28, 23	24, 22	24, 22
UMSC group success rate *n* (%)	15 (54%)	12 (50%)	11 (46%)
Placebo group success rate *n* (%)	6 (26%)	5 (23%)	3 (14%)
Difference in rates	28%	27%	32%
Chi-Square *p*-value	0.047	0.056	0.018

The mean HVAS Mood and sum indexed HVAS scores were both significantly improved following treatment in the UMSC group, whereas no significant differences over time were observed for the placebo group (Table 4). The mean HVAS Movement scores were also significantly improved following treatment in the UMSC group, whereas a significant difference from baseline was only observed at 3 months following treatment for the placebo group. There was a significant difference in HVAS Mood scores between treatment groups at 3 (*P* = 0.05) and 6 (*P* = 0.01) months, but there was no difference at 1 month (*P* = 0.06). There was a significant difference in HVAS sum scores between treatment groups 6 months (*P* = 0.03), but there was no difference at 1 month (*P* = 0.07).

**Table 4A:** Mean HVAS movement scores.

	**Baseline**	**1 Month**	**3 Months**	**6 Months**
UMSC mean	26.80	36.50^a^	40.64^a^	40.20^a^
Placebo mean	27.84	31.04	34.77^a^	32.65
Difference in means	−1.04	5.46	5.87	7.55
*p*-value (UMSC vs. control)	0.72	0.06	0.05	0.01

a *Significantly different from baseline within group*.

**Table 4B:** Mean HVAS mood scores.

	**Baseline**	**1 month**	**3 months**	**6 months**
UMSC mean	24.91	29.57^a^	30.28^a^	29.56^a^
Placebo mean	25.42	27.08	28.99	27.11
Difference in means	−0.51	2.49	1.30	2.44
*p*-value (UMSC vs. control)	0.77	0.16	0.48	0.19

a *Significantly different from baseline within group*.

**Table 4C:** Mean HVAS total scores.

	**Baseline**	**1 month**	**3 months**	**6 months**
UMSC mean	51.61	65.98^a^	70.80^a^	69.65^a^
Placebo mean	53.39	58.25	63.85	59.84
Difference in means	−1.78	7.73	6.95	9.81
*p*-value (UMSC vs. control)	0.68	0.07	0.12	0.03

a *Significantly different from baseline within group*.

**Table 4D:** Mean summed orthopedic examination score.

	**Baseline**	**1 month**	**3 months**	**6 months**
UMSC mean	13.25	11.57	11.60	11.60
Placebo mean	13.32	12.36	11.96	12.60
Difference in means	−0.07	−0.79	−0.36	−1.00
*p*-value (UMSC vs. control)	0.95	0.47	0.76	0.39

**Table 4E:** Mean Force Plate PVF on lame limb.

	**Baseline**	**1 month**	**3 months**	**6 months**
UMSC mean	85.56	91.56	88.22	91.33
Placebo mean	86.05	84.91	92.02	92.43
Difference in means	−0.49	6.66	−4.20	−1.10
*p*-value	0.87	0.02	0.22	0.69

### Orthopedic Examination and Force-Plate Analysis

The covariates of baseline values had no significant effects on the models for both orthopedic examination scores and force plate analysis values. No differences in PVF of the affected limb over time was observed in either treatment group (Table 4). Higher PVF was evident in the UMSC group when compared to placebo at 1 month following treatment (*P* = 0.02); however, there was no significant difference in PVF between groups at 3 or 6 months. No differences in the sum-indexed orthopedic scores were detected over time in either treatment group, or between treatment groups at equivalent time points.

## Discussion

This prospective clinical study of intra-articular UMSC therapy for canine elbow OA was both blinded and placebo controlled. According to the primary outcome variable based on owner assessment, a single intra-articular injection of allogenic UMSC reduced clinical signs of OA, when compared to a saline placebo. The positive effect appeared to persist throughout the 6 month duration of the study. None of the adverse events occurring in the UMSC group were found to be related to the treatment, which is of particular importance since this is the first study to assess the use of allogenic UMCS in a veterinary clinical setting.

Although the study detected statistical difference in success rates between treatment groups, our results should be interpreted in light of many factors when making clinical recommendations. The success rates based on CBPI in dogs treated with intra-articular UMSC ranged from 46 to 54%, which were approximately twice as high as the success rates in the placebo group. Whereas the response to treatment in this study may appear disappointing, we suspect low success rates found with both groups may be a reflection of the rigorous definition of success, rather than an inconsistent response to the treatment (22). Indeed, the response rates in our study were comparable to those reported in a study evaluating carprofen, which is considered to be a well-established and reliable therapeutic for dogs with OA (22).

Since quantifying clinical improvements directly related to therapeutics can be difficult in dogs with OA, numerous outcome measures have been developed. Our study used multiple tools for evaluating efficacy, but the proportion of dogs having success within a group based on CBPI was designated as the primary outcome. By reporting the percentage of dogs reaching a pre-determined level of improvement that is deemed clinically relevant, the results may give a greater sense of how likely an individual animal will respond favorably, when compared to reporting average scores for a group (22). This method is also less prone to being misinterpreted because of extreme outliers, which can have a large influence on mean or median values for pooled data (7, 18, 22). However, defining a clinically relevant target value, which defines success of the treatment, is highly subjective. In our study success was defined as a decrease in both PIS and PSS scores by at least one in dogs with baseline PIS and PSS of >2; this criterion was previously proven to be a robust method for comparing response to treatments for OA in dogs (22). It has also been suggested that owner-assessed outcomes such as CBPI are superior to veterinarian-based outcomes, because owners are able to observe dogs for much longer durations in familiar environments (18, 22). For these reasons, the FDA requires studies to utilize owner assessed outcome measures with classification of each animal as a success or failure.

Response to UMCS treatment appeared to sustain over the 6 months duration of follow-up. Based on our study design, the proportion of successful cases in the UMSC group would have approached the levels identified in the placebo group if the duration of response to UMCS was <6 months; however, success rates were approximately twice as high in the UMSC group throughout the follow-up period. It should be noted that a difference was not found at the 3 month time point. The lack of significance may have been caused by a Type-II statistical error, since a) the magnitude of difference between groups was similar for all post-treatment time points, and b) the number of subjects completing the study was below the original target set by the pre-study power analysis, suggesting the study was under-powered. A prolonged duration of effect is especially desirable because moderate to profound sedation is typically required for precise intra-articular injection, which may carry higher risks in an older population of dogs. Two previous clinical studies of autologous adipose-derived MSC in dogs with OA also found significant improvements for up to 6 months (3, 9). Collectively, these results suggest future studies should be designed to continue observations for >6 months in order to better define the expected duration of effects associated with stem cell therapy for OA.

Secondary outcome measures, including HVAS scores, force-platform gait analysis, and veterinarian-based examination scores, did not consistently identify differences between treatment groups. The HVAS scores improved in the UMSC group, but not the placebo group; however, significant differences between groups at equivalent time points were sporadic. Additionally, UMSC therapy did not appear to have an effect on veterinarian-based examination scores and force-platform gait analysis. Weak correlations between PVF and owner based assessments such as CBPI have been previously demonstrated (23), and the discrepancy of owner-based vs. veterinarian-based findings in this study could be explained by several possibilities. As discussed above, owner assessments are based on observing familiar subjects over a much longer period of time in their comfortable environment, and thus owners may have greater abilities to discern subtle responses. The owner questionnaires account for overall mobility and comfort, whereas the force-platform gait analysis and veterinarian examination more specifically evaluates the treated limb. The veterinarian assessment performed in this study encompassed parameters such as joint thickening and range of motion, and stem cell therapy is unlikely to reverse these types of chronic pathologic changes. Indeed, percutaneous delivery of stem cells within osteoarthritic joints are not expected to have any regenerative effect; rather, the proposed mechanism of action relates to modulation of inflammatory mediators (14). It is also possible that owner-bias played a role, where owners could be more hopeful for a positive response. Based on our force-platform and veterinarian-examination results, stem cell therapy alone may not have the ability to substantially improve lameness in dogs with chronic elbow OA, where structural changes of the joint may be a major contributor to limb dysfunction.

Stem cell therapy was very well-tolerated, and none of the dogs in the study developed adverse events that were likely to be related to UMSC treatment. Our findings are similar to studies in animals and humans reporting no adverse effects following intra-articular MSC treatment (5, 7, 24). The lack of side effects of MSC is attractive, because other intra-articular treatments for OA such as corticosteroids have reported risks associated with cytotoxicity, anaphylactic reactions or septic arthritis (25–27). Because of its safety, intra-articular UMSC may be particularly attractive for long term management of OA.

Despite some evidence showing MSC can be recognized by the host immune system (28), we did not observe any rejection response in our study. While promising, we only administered a single injection and potential adverse effects associated with repeated injections is unknown. The UMSC preparation in this study underwent rigorous quality control, including screening for infectious agents and use of strict sterile techniques. While there were two dogs in the UMSC group that developed neoplasia (hemangiosarcoma, lymphoma), the malignancies were not related to the treated joint. No animal studies have documented evidence of neoplasms at stem cell implantation sites, and the safety of intra-articular stem cell therapy in humans was recently confirmed in a large systematic literature review (29). It should be noted that most of the cited studies in that review were investigating autologous therapies, and the safety of allogenic UMSC needs further investigation.

Clinical efficacy of intra-articular MSC therapy for canine OA has been shown with both autologous and allogenic adipose-derived MSC in prospective, blinded clinical investigations (3, 4, 7, 9, 10). While it is not possible to accurately compare efficacy between studies, the umbilical source used in this study does offer several advantages over the described adipose-derived MSC. Harvest of autogenous adipose-derived MSC are associated with additional morbidity and cost, as surgical removal of fat is required. Cesarean section is a prerequisite for collecting UMSC; however, the decision to elect surgery is not in any way related to collection of the cells. It should be noted that allogenic adiposed-derived MSC may also be obtained ethically from “waste” tissue such as falciform fat during a laparotomy that was not performed specifically for the purposes of collecting MSC. The quantity and quality of MSC from adult bone marrow or adipose tissue can decline and/or become more variable with donor age (30, 31), whereas previous studies suggest UMCS may have greater proliferative and differentiating abilities than MCS derived from other sources (32).

There are several limitations to this study. We adopted less stringent definitions of success based on CBPI, as a reduction of PIS ≥2 (not 1) is more commonly used (22, 33); the optimal application of CBPI under different testing conditions remains to be determined. Although clinical improvement was documented, the results do not give any further insight into the mechanism of action of stem cell therapy for OA. The response to treatment was variable, and may have been influenced by several factors such as the severity of OA; for instance, dogs with complete loss of cartilage and severe OA may not have responded as well to this treatment when compared to those dogs with mild OA. Unfortunately, low case numbers precluded the ability to investigate factors contributing to a poor response. The vast majority of dogs had bilateral disease, yet the contralateral limb remained un-treated. Future studies should consider treatment of all joints assessed to be contributing to pain. Both the safety and efficacy of UMSC were not assessed for longer than 6 months, meaning longer term outcomes and adverse effects are unknown.

The results of this study demonstrated improvement of clinical signs related to elbow OA in dogs treated with intra-articular UMCS based on owner observations. Both objective and subjective veterinarian-based assessments were unable to distinguish between dogs treated with UMSC and a saline placebo, suggesting the effect of stem cell therapy may be limited. Encouragingly, intra-articular UMSC appear to have a prolonged duration of effect with minimal risk of complications. This treatment therefore may be a suitable alternative to conventional therapeutics for OA in dogs, but one should be aware of the relatively limited effect.

## Data Availability Statement

The raw data supporting the conclusions of this article will be made available by the authors, without undue reservation, to any qualified researcher.

## Ethics Statement

The animal study was reviewed and approved by University of Florida Institutional Animal Care and Use Committee. Written informed consent was obtained from the owners for the participation of their animals in this study.

## Author Contributions

AP, SK, KP, JY, ML-V, and DL contributed to study concept, literature review, data collection, data analysis, and manuscript preparation. JA, ST, AD, SC, and MJ contributed to data collection. All authors have read and approved the final submitted manuscript.

### Conflict of Interest

JY, ML-V, and KP were employed by the company Animal Cell Therapies. The remaining authors declare that the research was conducted in the absence of any commercial or financial relationships that could be construed as a potential conflict of interest.
